# Enhancing entanglement detection of quantum optical frequency combs via stimulated emission

**DOI:** 10.1038/s41598-019-41545-y

**Published:** 2019-03-25

**Authors:** Ievgen I. Arkhipov, Tai Hyun Yoon, Adam Miranowicz

**Affiliations:** 1 RCPTM, Joint Laboratory of Optics of Palacký University and Institute of Physics of CAS, Faculty of Science, Palacký University, 17.listopadu 12, 771 46 Olomouc, Czech Republic; 2 Center for Molecular Spectroscopy and Dynamics, Institute for Basic Science (IBS), Seoul, 02841 Republic of Korea; 3 Department of Physics, Korea University, Seoul, 02841 Republic of Korea; 4 Faculty of Physics, Adam Mickiewicz University, PL-61-614 Poznan, Poland

## Abstract

We investigate the performance of a certain nonclassicality identifier, expressed via integrated second-order intensity moments of optical fields, in revealing bipartite entanglement of quantum-optical frequency combs (QOFCs), which are generated in both spontaneous and stimulated parametric down-conversion processes. We show that, by utilizing that nonclassicality identifier, one can well identify the entanglement of the QOFC directly from the experimentally measured intensity moments without invoking any state reconstruction techniques or homodyne detection. Moreover, we demonstrate that the stimulated generation of the QOFC improves the entanglement detection of these fields with the nonclassicality identifier. Additionally, we show that the nonclassicality identifier can be expressed in a factorized form of detectors quantum efficiencies and the number of modes, if the QOFC consists of many copies of the same two-mode twin beam. As an example, we apply the nonclassicality identifier to two specific types of QOFC, where: (i) the QOFC consists of many independent two-mode twin beams with non-overlapped spatial frequency modes, and (ii) the QOFC contains entangled spatial frequency modes which are completely overlapped, i.e., each mode is entangled with all the remaining modes in the system. We show that, in both cases, the nonclassicality identifier can reveal bipartite entanglement of the QOFC including noise, and that it becomes even more sensitive for the stimulated processes.

## Introduction

One of the most striking features of quantum mechanics is quantum entanglement^1,2^, which accounts for the correlations between different parts of a system that cannot be described within the framework of classical physics. The development of the notion of the entanglement has led to the establishment of new branches of physics, e.g., quantum information theory^3^. Apart from its theoretical developments, entanglement has been already experimentally tested and exploited in quantum cryptography^4,5^, quantum communication^6–10^, quantum metrology^11^, quantum information processing^12^, and quantum machine learning^13,14^.

In order to implement quantum computation protocols, which utilize quantum properties of light, one needs highly-entangled quantum networks, e.g., continuous-variable (CV) cluster states^15^. These CV cluster states, which are mainly Gaussian states, can be constructed from multimode entangled light produced, e.g., in quantum-optical frequency combs (QOFCs)^16–20^. For quantum protocols based on discrete-variable cluster states, see, e.g., the review^21^.

On the other hand, the problem arises how to experimentally certify the entanglement of such multimode states. Methods have been proposed for verifying entanglement of CV states, in particular, Gaussian states. In most cases, these methods utilize various nonclassicality criteria for revealing the entanglement of such light. These include nonclassicality criteria based on, e.g., field-amplitude moments^22–30^, integrated intensity moments^24,31–33^, anomalous-field moments^34^, and the measured photocount histograms^35–38^, to name a few. Also, one may apply a CV-version of the Peres-Horodecki entanglement criterion to the reconstructed state^39,40^, or can use entanglement witnesses based on the separability eigenvalue equations^41,42^.

In a real experiment, it is desirable to have a simple, sensitive, and error robust tool for the entanglement identification of a detected QOFC. One of such methods includes a simple measurement of the mean and variance of the field intensity, which can be performed with the help of quadratic detectors and/or spectrometers. Of course, instead of intensities, one has to measure the mean value and variance of the quadratures of the measured fields. Nevertheless, the latter seems more complicated from the experimental point of view, as it requires balanced homodyne detection techniques along with the use of a spatial light modulator that has to shape the spectral profile of a local oscillator. Thus, naturally, one would prefer to resort to some nonclassicality identifiers (NIs) that just include the first- and second-order intensity moments of the measured fields.

For two-mode Gaussian states generated via spontaneous parametric processes, it has been recently shown in ref.^43^, that with the help of stimulated emission and by applying a certain NI, one can conclusively identify the entanglement of such states, by measuring their intensity moments up to the second order. We note, that a recent study in ref.^44^ has shown that with the measured variances of displaced quadratures one can reveal nonclassicality of any CV state.

In this work, motivated by the results in ref.^43^, we study a certain NI, which is based on the integrated second-order intensity moments, to show its applicability in identifying bipartite entanglement of the QOFC, which is generated in both spontaneous and stimulation parametric down-conversion processes. In particular, we consider two different scenarios: First, the QOFC consists of many independent two-mode twin beams, i.e., beams with non-overlapped spatial frequency modes. In the second scenario, the QOFC contains completely overlapped entangled spatial frequency modes, i.e., each mode is entangled with all the rest modes in the system. Most importantly, we show that with the help of the stimulation emission, one can enhance the sensitivity of the studied NI in the entanglement detection of the QOFC.

The paper is organized as follows. In Section *Theory* we briefly review a theory of spontaneous and stimulated down-converted QOFC. There, we also introduce a NI, which is expressed via integrated second-order intensity moments, and which we use throughout the paper. In Section *Entanglement identification of QOFC with spatially non-overlapping frequency modes*, we apply the NI to the QOFC that has non-overlapping entangled spatial frequency modes, i.e., independent two-mode twin beams. First, we study the performance of the NI for the two-mode case, and later we generalize it to any multimode bipartitions. Additionally, we consider the effect of stimulating fields on the performance of the NI. Section *Entanglement identification of QOFC with spatially overlapping frequency modes* is devoted to the case when the QOFC contains completely overlapped entangled spatial frequency modes. We show that for such QOFC, the considered NI also proves to be useful for the identification of multimode bipartite entanglement, and that the induced stimulation boosts the performance of a given NI. The conclusions are drawn in Section *Conclusions*.

## Theory

### General QOFC generated in spontaneous and stimulated down-conversion processes

First, we briefly review the dynamics of the quantum optical frequency comb generated in spontaneous parametric down-conversion (PDC) process, and that are driven by an intense classical optical frequency comb. Later, we focus on the dynamics of the QOFC that are generated in the stimulated PDC process, where the stimulating fields can also be classical optical frequency combs (COFCs).

The dynamics of the spontaneous PDC process is described by the following Hamiltonian in the interaction picture^45,46^.

1 $${H}_{I}=\int {\rm{d}}V{\chi }^{\mathrm{(2)}}{\hat{E}}_{p}^{-}{\hat{E}}_{s}^{+}{\hat{E}}_{i}^{+}+{\rm{h}}\,.\,{\rm{c}}\,.,$$

where ${\hat{E}}_{p}^{-}$ is the negative-frequency part of the electromagnetic field operator of the pump COFC field, and ${\hat{E}}_{{\rm{s}}}^{+}$ (${\hat{E}}_{{\rm{i}}}^{+}$) is the positive-frequency part of the electromagnetic field operator of the signal (idler) beam^45^; *χ*^(2)^ is the quadratic susceptibility of a nonlinear medium. The integration in Eq. (1) is performed over the medium volume *V*; h.c. stands for Hermitian conjugate.

In the parametric approximation, the pump field, which generates the pairs of entangled photons, can be treated classicaly. Thus, the operator ${\hat{E}}_{p}$, in Eq. (1), becomes a *c*-number. For the COFC pump field, which propagates along the *z*-axis, the electric-field amplitude can be written as follows^47^.

2 $$\begin{array}{rcl}{E}_{p}(t,z) & = & \sum _{m}A(t-m{\rm{\Delta }}T)\exp (\,-\,i{\omega }_{p}(t-m{\rm{\Delta }}T)-im\Delta {\varphi }_{ceo}+i{k}_{p}z)\\  & = & \exp (\,-\,i{\omega }_{p}t+i{k}_{p}z)\sum _{n=-\infty }^{\infty }{A}_{n}\exp (\,-\,in{\omega }_{r}t),\end{array}$$

where *ω*_*p*_ and *k*_*p*_ are the carrier frequency and wave vector of the pump field, respectively, *ω*_*r*_ is the angular frequency difference between the teeth of the COFC separated by the time interval Δ*T* = 2*π*/*ω*_*r*_. The carrier-envelope-offset phase is denoted by Δ*ϕ*_*ceo*_. The field amplitude *A*_*n*_ corresponds to the *n*th tooth of the COFC, i.e., to the *n*th frequency of the comb spectrum. For details regarding COFCs, we refer the reader to the appropriate literature, e.g., see refs^48,49^.

The operators of the electric fields for both signal and idler beams, which also propagate alone the *z*-axis, are quantized as follows

3 $${\hat{E}}_{j}^{+}=i\sum _{{k}_{j}}{\varepsilon }_{{k}_{j}}{\hat{a}}_{{k}_{j}}\exp (\,-\,i{\omega }_{{k}_{j}}t+i{k}_{j}z),\,j=s,i,$$

where ${\varepsilon }_{{k}_{j}}=\sqrt{2\pi \hslash {\omega }_{{k}_{j}}/{\mu }^{2}V}$ is the amplitude of the electric field per photon, *μ* is the frequency-dependent refractive index, and *V* is the quantization volume.

Combining now Eqs (1, 2), and (3), and assuming that the ideal phase-matching conditions are satisfied^45,50^, i.e., ${\omega }_{p}+n{\omega }_{r}={\omega }_{{k}_{s}}+{\omega }_{{k}_{i}}$, and *k*_*p*_ = *k*_*s*_ + *k*_*i*_, one arrives at the following Hamiltonian

4 $${H}_{I}=-\,\hslash \sum _{{k}_{s},{k}_{i}}{g}_{{k}_{s},{k}_{i}}{\hat{a}}_{{k}_{s}}{\hat{a}}_{{k}_{i}}+{\rm{h}}\,.\,{\rm{c}}\,.,$$

where ${g}_{{k}_{s},{k}_{i}}$ is a coupling constant proportional to both amplitude of the *n*th tooth of the COFC pump *A*_*n*_, and the nonlinear susceptibility *χ*^(2)^, and which is responsible for the coupling between the signal and idler modes with wave vectors *k*_*s*_ and *k*_*i*_, respectively. In what follows, without loss of generality, we assume that ${g}_{{k}_{s},{k}_{i}}$ is a real-valued parameter. The Hamiltonian in Eq. (4) describes the dynamics of the generated QOFC.

If we assume that there are *N* different spatial frequency modes in a QOFC, then, one can write down the Heisenberg equations for the boson operators, in Eq. (4), as follows

5 $$\frac{{\rm{d}}\hat{A}}{{\rm{d}}t}=i{M}\hat{A},$$

where $\hat{A}={({\hat{a}}_{1},{\hat{a}}_{1}^{\dagger },\ldots ,{\hat{a}}_{N},{\hat{a}}_{N}^{\dagger })}^{T}$ is a 2*N*-dimensional vector of the boson annihilation and creation operators, and *M* is a 2*N* × 2*N*-dimensional evolution matrix with elements ${g}_{{k}_{s},{k}_{i}}$.

The formal solution of Eq. (5) reads as

6 $$\hat{A}(t)=\exp (i{M}t)\hat{A}(0)={S}\hat{A}(0),$$

where we denoted the matrix exponential as *S*. Since we consider a system with a finite number of modes, the matrix *S* can always be presented as a 2*N* × 2*N*-dimensional matrix following the Jordan decomposition of the matrix *M*.

The knowledge of the quantum fields of the QOFC in Eq. (6) allows one to completely characterize QOFC state through the normally-ordered covariance matrix (CM) ${{A}}_{{\mathscr{N}}}$, which for an *N*-mode state is written as^51^:

7 $${{A}}_{{\mathscr{N}}}=(\begin{array}{cccc}{{\bf{A}}}_{1} & {{\bf{A}}}_{12} & \cdots  & {{\bf{A}}}_{1{\bf{N}}}\\ {{\bf{A}}}_{12}^{\dagger } & {{\bf{A}}}_{2} & \cdots  & \vdots \\ \vdots  & \vdots  & \ddots  & \vdots \\ {{\bf{A}}}_{1{\bf{N}}}^{\dagger } & \cdots  & \cdots  & {{\bf{A}}}_{{\bf{N}}}\end{array}).$$

where **A**_**k**_ and **A**_**jl**_ are the block 2 × 2 matrices:

8 $${{\bf{A}}}_{{\bf{k}}}=(\begin{array}{cc}{B}_{k} & {C}_{k}\\ {C}_{k}^{\ast } & {B}_{k}\end{array}),\,\begin{array}{ccc}{B}_{k} & = & \langle :{\rm{\Delta }}{\hat{a}}_{k}^{\dagger }{\rm{\Delta }}{\hat{a}}_{k}:\rangle ,\\ {C}_{k} & = & \langle :{\rm{\Delta }}{\hat{a}}_{k}^{2}:\rangle ,\end{array}$$

9 $${{\bf{A}}}_{{\bf{jl}}}=(\begin{array}{cc}{\bar{D}}_{jl}^{\ast } & {D}_{jl}\\ {D}_{jl}^{\ast } & {\bar{D}}_{jl}\end{array}),\,\begin{array}{rcl}{D}_{jl} & = & \langle :{\rm{\Delta }}{\hat{a}}_{j}{\rm{\Delta }}{\hat{a}}_{l}:\rangle ,\\ {\bar{D}}_{jl} & = & \langle :{\rm{\Delta }}{\hat{a}}_{j}^{\dagger }{\rm{\Delta }}{\hat{a}}_{l}:\rangle ,\end{array}$$

where $${\rm{\Delta }}\hat{O}=\hat{O}-\langle \hat{O}\rangle $$.

To include quantum thermal noise in the system, we employ a standard model based on the superpositions of the signal and noise^46,52^, where the inclusion of this kind of noise, with the mean noise photon-number 〈*n*〉, affects only the parameters *B*_*k*_ in Eq. (7), as *B*_*k*_ → *B*_*k*_ + 〈*n*_*k*_〉, and it leaves the other elements of the ${{A}}_{{\mathscr{N}}}$ unchanged.

In the case of stimulated PDC, i.e., when the generated QOFC is additionally seeded by stimulating coherent fields, the dynamics of the stimulating fields obeys the same equation of motion as in Eq. (6) for the boson operators, namely:

10 $${\rm{\Xi }}(t)={S}{\rm{\Xi }}(0),$$

where ${\rm{\Xi }}={({\xi }_{1},{\xi }_{1}^{\ast },\ldots ,{\xi }_{N},{\xi }_{N}^{\ast })}^{T}\in {{\mathbb{C}}}^{2N}$ is a complex vector of *N* stimulating coherent fields, and the matrix *S* is given in Eq. (6).

With the knowledge of the covariance matrix ${{A}}_{{\mathscr{N}}}$ and the vector of stimulating coherent fields Ξ(*t*), one can easily arrive at the generating function ${G}_{{\mathscr{N}}}$, as follows^51^:

11 $${G}_{{\mathscr{N}}}(\lambda )=\frac{1}{\sqrt{{\rm{\det }}\,{{A}{^{\prime} }}_{{\mathscr{N}}}}\prod _{j=1}^{n}{\lambda }_{j}}\exp (-\,\frac{1}{2}{{\rm{\Xi }}}^{\dagger }{{{A}{^{\prime} }}_{{\mathscr{N}}}}^{-1}{\rm{\Xi }}),$$

where ${\lambda }=({\lambda }_{1},\ldots ,{\lambda }_{n})\in {{\mathbb{R}}}^{n}$ is a real vector. The matrix ${{A}}_{{\mathscr{N}}}^{^{\prime} }={{A}}_{{\mathscr{N}}}+{{\rm{\Lambda }}}^{-1}$, where the matrix ${{\rm{\Lambda }}}^{-1}={\rm{diag}}(1/{\lambda }_{1},1/{\lambda }_{1},\ldots ,1/{\lambda }_{n},1/{\lambda }_{n})$.

The generating function ${G}_{{\mathscr{N}}}$ allows one to obtain statistical moments of integrated intensities of the fields and also their photon-number probability distribution function. The integrated-intensity moments ${\langle {W}_{1}^{{k}_{1}}\ldots {W}_{n}^{{k}_{n}}\rangle }_{{\mathscr{N}}}$ are obtained from^53^:

12 $${\langle {W}_{1}^{{k}_{1}}\ldots {W}_{n}^{{k}_{n}}\rangle }_{{\mathscr{N}}}={(-1)}^{{k}_{1}+\ldots +{k}_{n}}{\frac{{\partial }^{{k}_{1}+\ldots +{k}_{n}}{G}_{{\mathscr{N}}}({\rm{\lambda }})}{\partial {\lambda }_{1}^{{k}_{1}}\ldots \partial {\lambda }_{n}^{{k}_{n}}}|}_{{\lambda }_{1}=\ldots ={\lambda }_{n}=0}.$$

### Nonclassicality identifier expressed via intensity moments

One can write down various NIs, expressed in terms of integrated intensity moments of the first and second order. Such an NI can be derived either from a moments-matrix approach or, e.g., from a majorization theory^54^. At the same time, as recent studies indicate, the moments-matrix approach enables finding NIs with better performance than those derived from the majorization theory^38^. Below, we present two possible NIs based on second-order intensity moments, obtained from the moments-matrix approach, for the entanglement identification of bipartite states, i.e., the entanglement between two parts, denoted as signal and idler arms, as follows

13 $${E}_{1}={\langle {W}_{{\rm{s}}}^{2}\rangle }_{{\mathscr{N}}}{\langle {W}_{{\rm{i}}}^{2}\rangle }_{{\mathscr{N}}}-{\langle {W}_{{\rm{s}}}{W}_{{\rm{i}}}\rangle }_{{\mathscr{N}}}^{2},$$

and

14 $${E}_{2}={\langle {\rm{\Delta }}{{W}}_{{\rm{s}}}^{2}\rangle }_{{\mathscr{N}}}{\langle {\rm{\Delta }}{{W}}_{{\rm{i}}}^{2}\rangle }_{{\mathscr{N}}}-{\langle {\rm{\Delta }}{W}_{{\rm{s}}}{\rm{\Delta }}{W}_{{\rm{i}}}\rangle }_{{\mathscr{N}}}^{2},$$

where ${\rm{\Delta }}W=W-{\langle W\rangle }_{{\mathscr{N}}}$, and the moments ${\langle {W}_{{\rm{s}}}^{m}{W}_{{\rm{i}}}^{n}\rangle }_{{\mathscr{N}}}$ are given in Eq. (12). Whenever *E*_1_, *E*_2_ < 0, a bipartite state is considered to be nonclassical, particularly, can be entangled.

One of the most important properties of the NIs, *E*_1_, and *E*_2_, is that, the quantum detection efficiencies *η*_*s*_ and *η*_*i*_ of the signal- and idler-beam detectors, respectively, are factorized, i.e.,

15 $${E}_{j}({\eta }_{{\rm{s}}},{\eta }_{{\rm{i}}})={\eta }_{{\rm{s}}}^{2}{\eta }_{{\rm{i}}}^{2}{E}_{j}.$$

where on the r.h.s. of Eq. (15), the NIs *E*_*j*_ are for the ideal case *η*_*s*_ = *η*_*i*_ = 1. Therefore, in what follows, without loss of generality, we always assume that one uses ideal detectors.

As it has been already shown in ref.^51^, the NI *E*_1_ can be used for complete identification of nonclassicality of any mixed two-mode Gaussian state by utilizing a certain coherent displacement to the state. The NI *E*_2_, as our preliminary analysis has shown, does not possesses this universality. Nevertheless, for particular cases, such as multimode entangled Gaussian states, the NI *E*_2_ can be even more better than the NI *E*_1_. For instance, the NI *E*_2_ has a much simpler dependence on the number of modes, that can be utilized in more effective state reconstruction techniques based on this NI. Moreover, in the next sections, we show that for multimode QOFCs with either overlapping or non-overlapping spatial frequency modes, the NI *E*_2_ demonstrates a good performance in revealing of bipartite entanglement. Additionally, we show that the stimulation of a noiseless or low noisy QOFC boosts the performance of the NI *E*_2_. In other words, the NI *E*_2_ increases its negativity with the increasing intensity of stimulating fields. Hereafter, we denote NI *E*_2_ simply as *E*.

In general, the NI *E* can describe the nonclassicality of a bipartite state only qualitatively not in quantitative way. In order to relate this qualitative character of the NI *E* to a quantitative measure, we employ the method used in ref.^54^. Namely, we establish the correspondence between the NI *E* and the Lee’s nonclassicality depth *τ*, which is a good measure of nonclassicality^55^. The operational meaning of *τ* is that it determines the amount of thermal noise, one has to add into both arms of a bipartite system, to remove its nonclassicality. When considering a two-mode state, we relate *τ* to the least negative eigenvalue, taken with opposite sign, of the two-mode covariance matrix ${{A}}_{{\mathscr{N}}}$^56^. In this case, the condition *τ* > 0 is both necessary and sufficient for the nonclassicality of the two-mode state. For the multimode case, when considering multimode bipartitions involving *M* modes, we refer to *τ*_*M*_ as the *τ*-parametrized NI, ${E}_{{\tau }_{M}}$, that can be written as follows^33,54^:

16 $$\begin{array}{rcl}{E}_{{\tau }_{M}} & = & {\tau }_{M}^{4}+2{\tau }_{M}^{3}({\langle {W}_{{\rm{s}}}\rangle }_{{\mathscr{N}}}+{\langle {W}_{{\rm{i}}}\rangle }_{{\mathscr{N}}})\\  &  & +\,{\tau }_{M}^{2}({\langle {\rm{\Delta }}{{W}}_{{\rm{s}}}^{2}\rangle }_{{\mathscr{N}}}+{\langle {\rm{\Delta }}{W}_{{\rm{i}}}^{2}\rangle }_{{\mathscr{N}}}+4{\langle {W}_{{\rm{s}}}\rangle }_{{\mathscr{N}}}{\langle {W}_{{\rm{i}}}\rangle }_{{\mathscr{N}}})\\  &  & +\,2{\tau }_{M}({\langle {\rm{\Delta }}{W}_{{\rm{s}}}^{2}\rangle }_{{\mathscr{N}}}{\langle {W}_{{\rm{i}}}\rangle }_{{\mathscr{N}}}+{\langle {\rm{\Delta }}{W}_{{\rm{i}}}^{2}\rangle }_{{\mathscr{N}}}{\langle {W}_{{\rm{s}}}\rangle }_{{\mathscr{N}}})+{E}_{M},\end{array}$$

which determines the amount of thermal noise *τ*_*M*_, that one also has to add into both signal and idler arms of a bipartite *M*-mode state to erase the negativity of *E*_*M*_. In other words, the amount of nonclassicality *τ*_*M*_ is defined from the condition ${E}_{{\tau }_{M}}=0$. Importantly, in this case, the condition τ_*M*_ > 0 is no more necessary but only sufficient for the nonclassicality of a bipartite QOFC state. Since τ_*M*_ > 0 holds only when *E*_*M*_ < 0, according to Eq. (16). But the condition *E*_*M*_ < 0 is sufficient but not necessary for the nonclassicality identification. The reason why we resort to the *τ*_*M*_, derived from Eq. (16), and not from the multimode covariance matrix ${{A}}_{{\mathscr{N}}}$, is that for a large number of modes, *M* ≫ 1, the problem of finding the eigenvalues of a large-size matrix becomes computationally hard. Nevertheless, *τ*_*M*_ can serve as a nonclassicality quantifier for bipartite states of the QOFC.

## Entanglement identification of QOFC with spatially non-overlapping frequency modes

In this section, we apply the NI *E*, given in Eq. (14), to the QOFC that consists of non-overlapping spatial frequency modes, i.e., any spatial frequency mode *k*_*s*_ is entangled with only one given mode *k*_*i*_. In other words, this QOFC is comprised of many independent two-mode twin beams. We note that, in general, the down-converted frequency modes constituting QOFC, which are generated by different frequencies of the COFC pump, can overlap. The latter case is considered in the next section. Here, instead, we consider the case when such overlapping does not occur. Such QOFC has already been experimentally realized in ref.^57^, and, for example, in cavity-enhanced spontaneous PDC^58–62^. Additionally, to make our analysis simpler, we will first focus on a two-mode case and, then, we will proceed to the multimode scenario.

### Two-mode entanglement

#### Spontaneous PDC

For the QOFC, with non-overlapping spatial frequency modes, which is generated in a spontaneous PDC, the boson operators of the signal and idler modes of a two-mode twin beam, produced by the *n*th tooth of the COFC pump, according to Eq. (6), acquires the following form

17 $$\begin{array}{rcl}{\hat{a}}_{{\rm{s}},n}(t) & = & \cosh ({g}_{n}t){\hat{a}}_{{\rm{s}},n}(0)+i\,\sinh ({g}_{n}t){\hat{a}}_{{\rm{i}},n}^{\dagger }(0),\\ {\hat{a}}_{{\rm{i}},n}(t) & = & \cosh ({g}_{n}t){\hat{a}}_{{\rm{i}},n}(0)+i\,\sinh ({g}_{n}t){\hat{a}}_{{\rm{s}},n}^{\dagger }(0).\end{array}$$

For simplicity, we drop the subscript *n* in the boson operators.

In that case, the covariance matrix ${{A}}_{{\mathscr{N}}}$, in Eq. (7), of the whole QOFC is factorized on a set of independent 4 × 4 matrices, each corresponding to a two-mode twin beam. The nonzero elements of a given two-mode covariance matrix read as follows:

18 $${B}_{j}={B}_{{\rm{p}}}+\langle {n}_{j}\rangle ,\,{D}_{{\rm{si}}}=i\sqrt{{B}_{{\rm{p}}}({B}_{{\rm{p}}}+1)},\,j={\rm{s}},{\rm{i}},$$

where ${B}_{{\rm{p}}}={\sinh }^{2}gt$ is the mean photon number of entangled pairs, and 〈*n*_*j*_〉 is the mean thermal noise photon-number in *j*th mode.

Combining now together Eqs (18, 11) and (12), the NI *E*, in Eq. (14), can be written as

19 $${E}^{{\rm{sp}}}=({B}_{{\rm{s}}}{B}_{{\rm{i}}}-|{D}_{{\rm{si}}}{|}^{2})({B}_{{\rm{s}}}{B}_{{\rm{i}}}+|{D}_{{\rm{si}}}{|}^{2}),$$

where superscript sp in *E*^sp^ accounts for spontaneous PDC.

**Figure 1:**
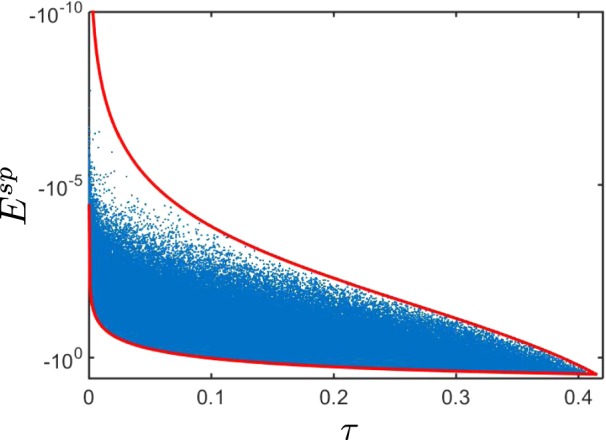
Nonclassicality identifier *E*^sp^, given in Eq. (19), versus the Lee’s nonclassicality depth *τ* for randomly generated 10^6^ states of a mixed two-mode twin beam using a Monte-Carlo simulation. Each point on the graph denotes a certain mixed twin beam state for which *E*^sp^ and *τ* are calculated. Upper and lower red solid curve intersect the line *τ* = 0 only at the point *E*^sp^ = 0.

The expression in the first bracket, in Eq. (19), is a Fourier determinant of the normally-ordered characteristic function of the two-mode twin beam^53^. Hence, when this determinant is negative, the Glauber-Sudarshan *P* function, which is the Fourier transform of the normally-ordered characteristic function, fails to be a classical distribution function^45,46^. The latter serves as a definition of the nonclassicality and, therefore, determines the entanglement of the twin beam state. Therefore, whenever a twin beam is entangled, *E*^sp^ always attains negative values. As such, *E*^sp^ becomes a genuine NI for the two-mode twin beams. Figure 1 shows the dependence of the NI *E*^sp^ on the Lee’s nonclassicality depth *τ*. This graph indicates that *E*^sp^ is a nonclassicality monotone for any mixed two-mode twin beam, i.e., whenever τ > 0, then *E*^sp^ < 0.

For pure two-mode twin beams, the NI *E*^sp^ attains a simple form

20 $${E}^{{\rm{sp}}}=-\,{B}_{{\rm{p}}}^{2}(2{B}_{{\rm{p}}}+1).$$

Hence, the more intense is the twin beam the larger is its entanglement and, thus, the greater is the negativity of *E*^sp^.

#### Stimulated PDC

In a stimulated PDC process, the generated twin beam at the output of a nonlinear crystal contains a nonzero coherent part due to the presence of stimulating coherent fields. The stimulation process of the twin beams, generated by a COFC pump, can be realized by another COFC that seeds both signal and idler fields. The dynamics of such stimulating fields, which stimulate the *n*th twin beam, as given in Eq. (17), reads according to Eq. (10), as follows

21 $$\begin{array}{rcl}{\xi }_{{\rm{s}}}(t) & = & \cosh (gt){\xi }_{{\rm{s}}}(0)+i\,\sinh (gt){\xi }_{{\rm{i}}}^{\ast }\mathrm{(0),}\\ {\xi }_{{\rm{i}}}(t) & = & \cosh (gt){\xi }_{{\rm{i}}}(0)+i\,\sinh (gt){\xi }_{{\rm{s}}}^{\ast }(0).\end{array}$$

Hereafter, for simplicity, we assume that the stimulation process is performed by a seeding COFC that stimulates only the signal field, i.e., *ξ*_i_(0) = 0.

**Figure 2:**
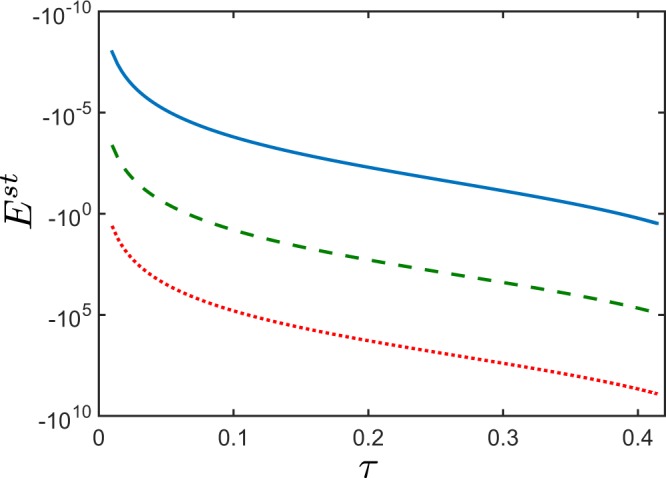
Nonclassicality identifier *E*^st^, according to Eq. (22), versus the nonclassicality depth *τ* for pure stimulated twin beams. The stimulation is applied only in the signal field. The NI *E*^st^ when the stimulating signal field is: *ξ*_s_ = 0 (blue solid curve), *ξ*_s_ = 10 (green dashed curve), and *ξ*_s_ = 100 (red dotted curve). The mean photon-number of pairs *B*_p_ ∈ (0, 1]. For a given value of *τ*, by increasing the intensity of the stimulating field, the negativity of *E*^st^ also increases.

For pure states, the NI *E*, then, acquires the following form

22 $${E}^{{\rm{st}}}=-\,{B}_{{\rm{p}}}^{2}(2{B}_{{\rm{p}}}+1)-4{B}_{{\rm{p}}}^{2}|{\xi }_{{\rm{s}}}(0){|}^{2}[|{\xi }_{{\rm{s}}}(0){|}^{2}({B}_{{\rm{p}}}+1)+\frac{3}{2}{B}_{{\rm{p}}}+1],$$

where the first term accounts for the negativity of *E*^st^ due to spontaneous emission, and the second term corresponds to stimulated emission. For a given value of *τ*, *E*^st^ increases its negative value with the increasing amplitude of stimulating field *ξ*_s_ (see Fig. 2). This means that, the stronger is the stimulating field *ξ*_s_, the more negative is *E*^st^. Moreover, as indicated by Eq. (22), *E*^st^ is independent of the phase of the stimulating field *ξ*_s_ and it depends solely on the coherent field intensity.

### Multimode bipartite entanglement

Now, we apply the NI *E*, as denoted by *E*_*M*_, for certifying bipartite entanglement of the multimode QOFC, consisting of *M* independent two-mode twin beams. By performing bipartition of a multimode twin beam such that all the signal modes belong to the signal arm, and all the idler modes to the idler arm, *E*_*M*_, then, can be written as follows

23 $${E}_{M}=\sum _{n=1}^{M}{\langle {\rm{\Delta }}{W}_{{\rm{s}},n}^{2}\rangle }_{{\mathscr{N}}}\sum _{n=1}^{M}{\langle {\rm{\Delta }}{W}_{{\rm{i}},n}^{2}\rangle }_{{\mathscr{N}}}-\,{(\sum _{n=1}^{M}{\langle {\rm{\Delta }}{W}_{{\rm{s}},n}{\rm{\Delta }}{W}_{{\rm{i}},n}\rangle }_{{\mathscr{N}}})}^{2},$$

where

24 $$\begin{array}{rcl}{\langle {\rm{\Delta }}{W}_{a,n}^{2}\rangle }_{{\mathscr{N}}} & = & {B}_{a,n}({B}_{a,n}+\mathrm{2|}{\xi }_{a,n}(t{)|}^{2}),\\ {\langle {\rm{\Delta }}{W}_{{\rm{s}},n}{\rm{\Delta }}{W}_{{\rm{i}},n}\rangle }_{{\mathscr{N}}} & = & -2{\rm{Re}}[{\xi }_{{\rm{s}},n}(t){\xi }_{{\rm{i}},n}(t){D}_{{\rm{si}},n}^{\ast }]-|{D}_{{\rm{si}},n}{|}^{2}.\end{array}$$

for *a* = s, *i*, *n* = 1, …, *M*. Note that we have assumed a general stimulated PDC process in the derivation of Eq. (24).

If the system is comprised of *M* copies of the same two-mode twin beam with the same stimulation, one then attains

25 $${E}_{M}={M}^{2}E,$$

where *E* is the NI for a two-mode twin beam copy, which we considered earlier. Thus, the number *M* of copies of the same two-mode twin beam serves as a coherent multiplier of the negativity of *E*_*M*_.

#### Spontaneous PDC

For a multimode spontaneous PDC process, the NI *E*_*M*_, given in Eq. (23), attains the form

26 $${E}_{M}^{{\rm{s}}p}=\sum _{n=1}^{M}{B}_{{\rm{s}},n}^{2}\sum _{n=1}^{M}{B}_{{\rm{i}},n}^{2}-{(\sum _{n=1}^{M}|{D}_{{\rm{si}},n}{|}^{2})}^{2}.$$

For the symmetric case, when $\sum _{n=1}^{M}{B}_{{\rm{s}},n}^{2}=\sum _{n=1}^{M}{B}_{{\rm{i}},n}^{2}$, Eq. (26) reduces to

27 $${E}_{M}^{{\rm{sp}}}=\sum _{n=1}^{M}({B}_{{\rm{s}},n}^{2}-|{D}_{{\rm{si}},n}{|}^{2})\sum _{n=1}^{M}({B}_{{\rm{s}},n}^{2}+|{D}_{{\rm{si}},n}{|}^{2}).$$

It is clearly seen that the first sum in Eq. (27) is the sum of the Fourier determinants of the normally-ordered characteristic functions of each two-mode twin beam, which is in analogy to Eq. (19). For the symmetric case, ${E}_{M}^{{\rm{sp}}}$ becomes the sum of the nonclassicality monotones of each two-mode twin beam. If the *n*th two-mode twin beam is entangled, then it contributes to the total negativity of ${E}_{M}^{{\rm{sp}}}$. Hence, the larger is the number of entangled two-mode twin beams in the system, the larger is the negativity of ${E}_{M}^{{\rm{sp}}}$. The number *M* of modes serves as a coherent amplifier for the entanglement detection of ${E}_{M}^{{\rm{sp}}}$, also due to the last positive sum in Eq. (27).

For pure multimode twin beams, ${E}_{M}^{{\rm{sp}}}$ in Eq. (27) can be written as follows

28 $${E}_{M}^{{\rm{sp}}}=-\,\sum _{n=1}^{M}{B}_{{\rm{p}},n}(\sum _{n=1}^{M}{B}_{{\rm{p}},n}(2{B}_{{\rm{p}},n}+1)).$$

**Figure 3:**
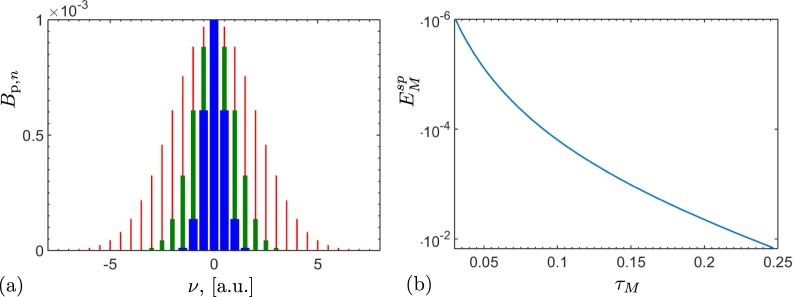
(**a**) Various spectral densities of a noiseless QOFC with *M* = 200 twin beams with the mean photon numbers of pairs obeying the Gaussian distribution ${B}_{{\rm{p}},n}\equiv {10}^{-3}\exp (\,-\,{\nu }_{n}^{2}\mathrm{/2}{\sigma }^{2})$, with *σ* = 2 (red narrow bars), *σ* = 1 (green thicker bars), and *σ* = 0.5 (blue thickest bars). Each tooth in panel (**a**) represents a twin beam with two spatially-separated modes of the same frequency. (**b**) NI ${E}_{M}^{{\rm{sp}}}$ for noiseless QOFC, according to Eq. (28), versus the nonclassicality depth *τ*_*M*_, defined in Eq. (16), for different spectra *B*_p,*n*_ shown in panel (**a**) but with *σ* in the range *σ* ∈ [0, 5]. The larger is the spectral density of the QOFC, the larger is the nonclassicality depth *τ*_*M*_, and, thus, the more negative is ${E}_{M}^{{\rm{sp}}}$.

Figure 3 shows the dependence of ${E}_{M}^{{\rm{sp}}}$ on the Lee’s nonclassicality depth *τ*_*M*_, defined in Eq. (16), for different spectral distributions of the QOFC displayed in Fig. 3(a). Therefore, the larger is the spectral energy of the QOFC, i.e., the larger is the number of the two-mode twin beams, the larger is the nonclassicality depth *τ*_*M*_, and, as a result, the larger is the negativity of ${E}_{M}^{{\rm{sp}}}$.

#### Stimulated PDC

Now, we consider stimulated PDC, when each *n*th signal beam is stimulated in the signal arm by a coherent field *ξ*_s,*n*_. Then, the NI *E*_*M*_, in Eq. (23), for a bipartite *M*-mode twin beam state is

29 $${E}_{M}^{{\rm{st}}}={E}_{M}^{{\rm{sp}}}-\sum _{n=1}^{M}|{\xi }_{{\rm{s}},n}{|}^{2}{f}_{n},$$

where ${E}_{M}^{{\rm{sp}}}$ is given in Eq. (26), *f*_*n*_ is a function of both number of the modes *M* and elements of covariance matrix ${{A}}_{{\mathscr{N}}}$ of the multimode QOFC. Whenever each two-mode twin beam of a given QOFC is entangled, then *f*_*n*_ ≥ 0. Meaning that, in this case, stimulating fields improve the performance of ${E}_{M}^{{\rm{st}}}$.

**Figure 4:**
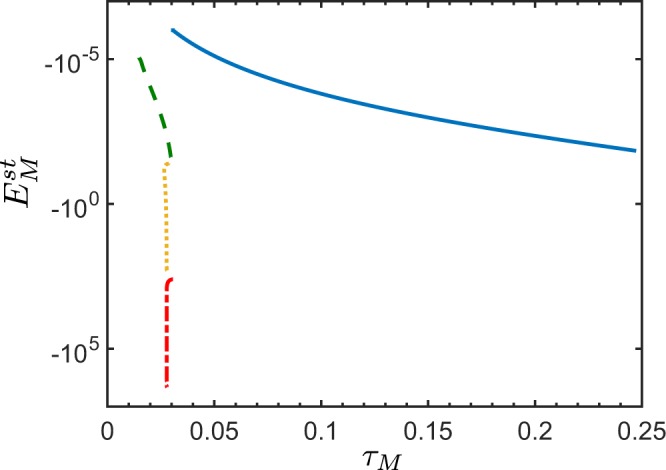
Nonclassicality identifier ${E}_{M}^{{\rm{st}}}$ versus nonclassicality depth *τ*_*M*_ for a given bipartition of the stimulated noiseless QOFC, where both signal and idler arms contain *M* = 200 modes, for different QOFC spectra at ${B}_{{\rm{p}},n}\equiv {10}^{-3}\exp (\,-\,{\nu }_{n}^{2}\mathrm{/2}{\sigma }^{2})$, *σ* ∈ [0, 5]. The twin beams are stimulated only in the signal modes. The coherent stimulating field *ξ*_s_ in the signal arm is set to: *ξ*_s_ = 0 (blue solid curve), *ξ*_s_ = 1 (green dashed curve), *ξ*_s_ = 10 (orange dotted curve), and *ξ*_s_ = 100 (red dash-dotted curve). The real spectra of the stimulating coherent field that stimulates the *n*th signal mode is $|{\xi }_{{\rm{s}},n}|\equiv |{\xi }_{{\rm{s}}}|\exp (\,-\,{\nu }_{n}^{2}/2{\sigma }^{2})$. With the increasing intensities of the stimulating fields, the sensitivity to noise of ${E}_{M}^{{\rm{st}}}$ also increases. Moreover, for very large stimulating fields, the amount of noise *τ*_*M*_, needed to make ${E}_{M}^{{\rm{st}}}$ positive, becomes independent of the intensity of the coherent field.

As in the two-mode case, this stimulation leads to the enhancement of the NI ${E}_{M}^{{\rm{st}}}$ (see Fig. 4). At the same time, as Fig. 4 shows, ${E}_{M}^{{\rm{st}}}$ becomes very sensitive to noise. Namely, by increasing the intensities of the stimulating fields, ${E}_{M}^{{\rm{st}}}$ becomes more negative, but at the expense of losing the tolerance to larger noise.

We note that, although the application of the NI *E*_*M*_ in Eq. (23) to the multimode twin beam seems straightforward, in practical situations, to separate the signal and idler modes might be difficult. Thus, the following problem arises: How to perform an appropriate bipartition that *E*_*M*_ can detect conclusively the modes entanglement. In this case, one needs to implement all possible bipartitions for *E*_*M*_ to reveal the maximal total entanglement of the multimode twin beam state.

## Entanglement identification of QOFC with spatially overlapping frequency modes

In this section, we discuss another type of a QOFC, namely, when the signal mode of a twin beam generated by the *n*th tooth of the COFC pump spatially overlaps with the signal or idler modes of the other twin beams produced by different or the same OFC teeth. As a result, one cannot simply divide such QOFC into a set of independent two-mode twin beams, as it was the case discussed in Section *Entanglement identification of QOFC with spatially non-overlapping frequency modes*.

Now, we consider the following interaction Hamiltonian

30 $$\hat{H}=-\,\hslash g\sum _{{k}_{s},{k}_{i}}{\hat{a}}_{{k}_{s}}{\hat{a}}_{{k}_{i}}+{\rm{h}}.{\rm{c}}.,$$

where we assume that the coupling strength *g* for each generated entangled pair is the same and real. As Eq. (30) implies, any spatial frequency mode *k*_*s*_ is equally coupled to various spatial frequency modes *k*_*i*_. Meaning that a given *k*_*s*_ mode can contain photons that are simultaneously entangled to different modes *k*_*i*_.

For this case, when the Hamiltonian in Eq. (30) contains *N* different spatial frequency modes, the evolution 2*N* × 2*N* matrix, in Eq. (5), takes the form ${M}=g{L}_{1}\otimes {L}_{2}$, where

31 $${L}_{1}=(\begin{array}{ll}0 & 1\\ -1 & 0\end{array}),\,{L}_{2}=(\begin{array}{llll}0 & 1 & \ldots  & 1\\ 1 & 0 & \ldots  & 1\\ \vdots  & \ldots  & \ddots  & \vdots \\ 1 & \ldots  & 1 & 0\end{array}),$$

and *L*_2_ is a *N* × *N* hollow matrix of ones, i.e., all its elements equal one, except the main diagonal elements which are zero.

The elements of the symmetric exponential matrix *S* = exp(*iMt*), in Eq. (6), after straightforward but some algebra, can be found as follows

32 $$\begin{array}{rcl}{S}_{j,j} & = & \frac{1}{2N}(\cosh \,[(N-1)gt]+(N-1)\cosh \,[gt]),\\ {S}_{j,j+1} & = & \frac{i}{2N}(\sinh \,[(N-1)gt]-(N-1)\sinh \,[gt]),\\ {S}_{j,2k+1} & = & \frac{1}{2N}(\cosh \,[(N-1)gt]-(N-1)\cosh \,[gt]),\\ {S}_{j,2k+2} & = & \frac{i}{2N}(\sinh \,[(N-1)gt]+(N-1)\sinh \,[gt]),\end{array}$$

for *j* = 1, …, *N*, and *k* = 1, …, *N* − 1. Having the matrix *S*, we can immediately obtain the normally-ordered covariance matrix ${{A}}_{{\mathscr{N}}}$ in Eq. (7). Thus, by combining Eqs (7) and (32), we obtain the elements of the matrix ${{A}}_{{\mathscr{N}}}$, which read as follows

33 $$\begin{array}{rcl}{B}_{{\rm{p}},j} & = & \frac{1}{2N}(\cosh \,[2(N-1)gt]+(N-1)\cosh \,[2gt])-\frac{1}{2},\\ {C}_{j} & = & \frac{i}{2N}(\sinh \,[2(N-1)gt]-(N-1)\sinh \,[2gt]),\\ {D}_{jk} & = & \frac{i}{2N}(\sinh \,[2(N-1)gt]+(N-1)\sinh \,[2gt]),\\ {\bar{D}}_{jk} & = & \frac{1}{N}({\sinh }^{2}[(N-1)gt]-{\sinh }^{2}[gt]),\end{array}$$

for *j*, *k* = 1, …, *N*. Since the parameter *B*_p,*j*_ does not account for mean photon-numbers of pairs anymore, as it was in Section *Entanglement identification of QOFC with spatially non-overlapping frequency modes*, we will call it simply as the mean photon number of vacuum fluctuations of the spatial frequency mode *j*. Considering the stimulation process, where each frequency mode of the QOFC is seeded by a coherent field *ξ*_*j*_, the dynamics of these stimulating fields obeys Eq. (10) with the matrix *S*, given in Eq. (32).

### Two-mode entanglement

#### Spontaneous PDC

For two-mode entanglement of the QOFC generated in spontaneous PDC with spatially overlapped frequency modes, the NI *E*, after applying Eq. (14), reads as follows

34 $${E}_{jk}^{{\rm{sp}}}=({B}_{j}^{2}+|{C}_{j}{|}^{2})({B}_{\phantom{j}\,k}^{2}+|{C}_{\phantom{j}k}{|}^{2})-{(|{D}_{jk}{|}^{2}+|{\bar{D}}_{jk}{|}^{2})}^{2},$$

where *B*_*j*_ = *B*_p,*j*_ + 〈*n*_*j*_〉 with the mean thermal noise photon-number 〈*n*_*j*_〉 in the *j*th mode, and *B*_p,*j*_, *C*_*j*_, *D*_*jk*_, and ${\bar{D}}_{jk}$ are given in Eq. (33). For simplicity, we will drop the subscripts in Eq. (34), as we are interested only in two-mode states.

**Figure 5:**
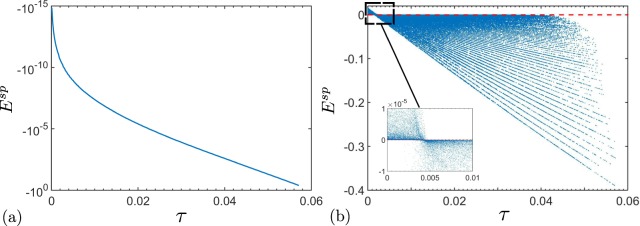
(**a**) Nonclassicality identifier *E*^sp^ versus nonclassicality depth *τ* for any two spatial frequency modes of the noiseless QOFC without stimulation. (**b**) The same as in panel (**a**) but for the mixed two-mode state. The total number of spatial frequency modes of the generated QOFC is *N* = 100, and the mean photon number of vacuum fluctuations and thermal noise photon number in each spatial frequency mode is *B*_p,*j*_, 〈*n*_*j*_〉 ∈ [0, 1], respectively. In general, *E*^sp^ is not a monotone of *τ*. Nevertheless, whenever *τ* > 0.5/*N*, *E*^sp^ is always a monotone of *τ*.

For noiseless QOFC, there is one-to-one correspondence between the NI *E*^sp^ and the Lee’s nonclassicality depth *τ* [see Fig. 5(a)]. The latter means that with the increasing entanglement between two given modes of the QOFC, the negativity of *E*^sp^ also increases.

In general, *E*^sp^ in Eq. (34) fails to detect entanglement between two different spatial frequency modes for noisy QOFC. Namely, as Fig. 5(b) indicates, there is a region of nonclassicality and entanglement, where the NI *E*^sp^ is positive. Nevertheless, as our numerical findings show, for a two-mode state with large nonclassicality, i.e., with large values of *τ*, the NI *E*^sp^ is always a monotone of *τ*. Moreover, for large number of modes, *N* ≫ 1, generated in QOFC, there is a bound for *τ*. Namely, whenever *τ* > 0.5/*N*, the NI *E*^sp^ is always negative [see Fig. 5(b)]. In other words, with the increasing *N* number of the modes in the QOFC, the NI *E*^sp^ tends to become a genuine monotone of *τ*. We note that the value *τ* = 0.5 is a maximal value of the nonclassicality depth, which can be reached by a Gaussian state.

#### Stimulated PDC

For the stimulated QOFC, the variances ${\langle {\rm{\Delta }}{W}_{j}^{m}{\rm{\Delta }}{W}_{k}^{n}\rangle }_{{\mathscr{N}}}$ of the integrated intensity moments, defined in Eq. (12), read as follows

35 $$\begin{array}{rcl}{\langle {\rm{\Delta }}{W}_{j}^{2}\rangle }_{{\mathscr{N}}} & = & {B}_{j}^{2}+|{C}_{j}{|}^{2}+2{B}_{j}^{2}|{\xi }_{j}{|}^{2}+2{\rm{Re}}[{C}_{j}{\xi }_{j}^{\ast 2}],\\ {\langle {\rm{\Delta }}{W}_{j}{\rm{\Delta }}{W}_{k}\rangle }_{{\mathscr{N}}} & = & |{D}_{jk}{|}^{2}+|{\bar{D}}_{jk}{|}^{2}+2{\rm{Re}}[{D}_{jk}{\xi }_{j}^{\ast }{\xi }_{k}^{\ast }]+2{\rm{Re}}[{\bar{D}}_{jk}{\xi }_{j}{\xi }_{k}^{\ast }].\end{array}$$

**Figure 6:**
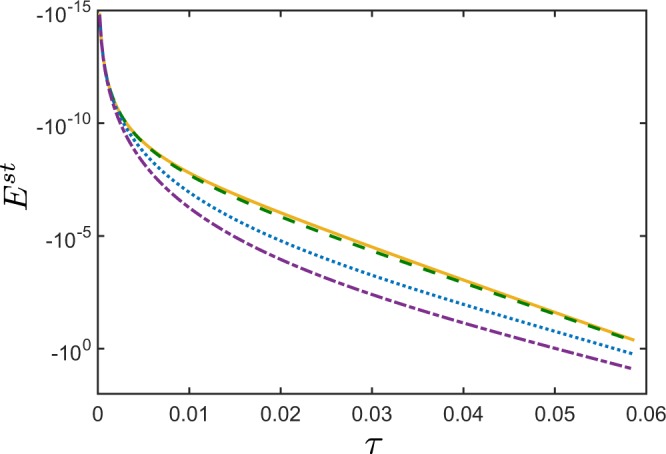
Nonclassicality identifier *E*^st^ versus nonclassicality depth *τ* for any two spatial frequency modes of the stimulated noiseless QOFC. The stimulation is applied only in one spatial frequency mode that does not belong to the given two-mode state. The amplitude of the stimulating field is: *ξ* = 0 (yellow solid curve), *ξ* = 10 (green dashed curve), *ξ* = 50 (blue dotted curve), *ξ* = 100 (violet dash-dotted curve). The total number of spatial frequency modes of the generated QOFC is *N* = 100, and the mean photon number of vacuum fluctuations in each spatial frequency mode is *B*_p,*j*_ ∈ [0, 1]. The NI *E*^st^ is independent on the phase of *ξ*. For a given value of *τ*, *E*^st^ exhibits larger negative values for larger stimulating-field amplitudes.

For noiseless QOFC, even when seeding either the *k*th mode that does not belong to a given two-mode state, the negativity of the NI *E*^st^ increases with the increasing seeding field *ξ*_*k*_ (see Fig. 6). Moreover, *E*^st^ is independent on the phase of the stimulating signal field *ξ*_*k*_.

### Multimode bipartite entanglement

For a bipartite state that contains *M* modes in both signal and idler arms, the applied *E*_*M*_, given in Eq. (14), takes the following form

36 $${E}_{M}={\langle {(\sum _{j=1}^{M}{\rm{\Delta }}{W}_{\mathrm{1,}j})}^{2}\rangle }_{{\mathscr{N}}}{\langle {(\sum _{j\mathrm{=1}}^{M}{\rm{\Delta }}{W}_{\mathrm{2,}j})}^{2}\rangle }_{{\mathscr{N}}}-{\langle \sum _{j=1}^{M}{\rm{\Delta }}{W}_{\mathrm{1,}j}\sum _{k=1}^{M}{\rm{\Delta }}{W}_{\mathrm{2,}k}\rangle }_{{\mathscr{N}}}^{2}.$$

We note that, compared to Eqs (23, 36) has a more complicated form due to the simultaneous presence of auto- and cross-correlations in both arms denoted as *W*_1_ and *W*_2_. Since each of those arms contains *M* modes which are also entangled. In other words, each term in Eq. (36) consists of a sum of different single- and two-mode integrated intensity moments, given in Eq. (35). For a symmetric system, i.e., when all the modes are statistically equivalent, the terms in Eq. (36) can be simplified as follows

37 $$\begin{array}{rcl}{\langle {(\sum _{j=1}^{M}{\rm{\Delta }}{W}_{1,j})}^{2}\rangle }_{{\mathscr{N}}} & = & M{\langle {\rm{\Delta }}{W}_{j}^{2}\rangle }_{{\mathscr{N}}}+M(M-1){\langle {\rm{\Delta }}{W}_{j}{\rm{\Delta }}{W}_{k}\rangle }_{N},\\ {\langle \sum _{j=1}^{M}{\rm{\Delta }}{W}_{1,j}\sum _{k=1}^{M}{\rm{\Delta }}{W}_{\mathrm{2,}k}\rangle }_{{\mathscr{N}}} & = & {M}^{2}{\langle {\rm{\Delta }}{W}_{j}{\rm{\Delta }}{W}_{k}\rangle }_{{\mathscr{N}}},\end{array}$$

where ${\langle {\rm{\Delta }}{W}_{j}^{2}\rangle }_{{\mathscr{N}}}$ and ${\langle {\rm{\Delta }}{W}_{j}{\rm{\Delta }}{W}_{k}\rangle }_{{\mathscr{N}}}$ are given in Eq. (35).

#### Spontaneous PDC

**Figure 7:**
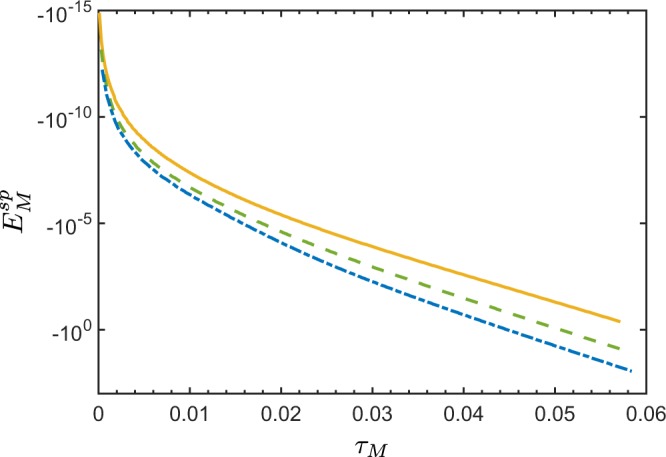
Nonclassicality identifier ${E}_{M}^{{\rm{sp}}}$ versus nonclassicality depth *τ*_*M*_ for a certain bipartition of the fields of a noiseless QOFC, where each part contains the following number of spatially-frequency modes: *M* = 1 (yellow solid curve), *M* = 3 (green dashed curve), and *M* = 6 (blue dash-dotted curve). The total number of spatial frequency modes of the generated QOFC is *N* = 100, and the mean photon number of vacuum fluctuations in each spatial frequency mode is *B*_p,*j*_ ∈ [0, 1]. The NI ${E}_{M}^{{\rm{st}}}$ displays a larger negativity when one includes more spatial frequency modes in a given bipartition.

In the case of the spontaneous PDC, the negativity of the NI ${E}_{M}^{{\rm{sp}}}$ is increasing with the increasing number *M* of the modes involved in a given bipartition (see Fig. 7). This means, that by inserting another pair of the spatial frequency modes into the bipartition, one boosts the sensitivity of ${E}_{M}^{{\rm{sp}}}$ in the entanglement detection of a given state.

#### Stimulated PDC

**Figure 8:**
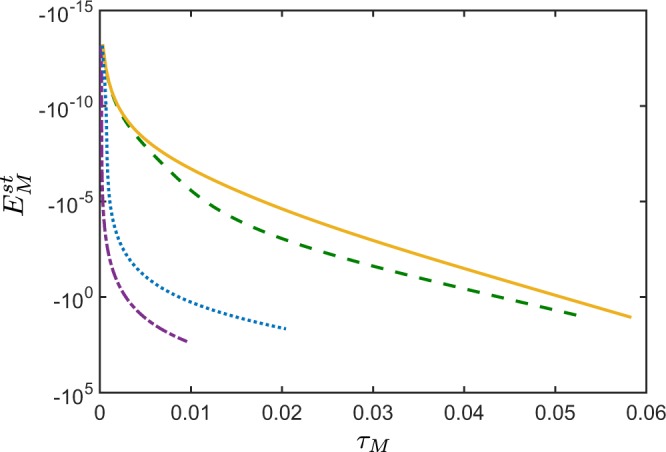
Nonclassicality identifier ${E}_{M}^{{\rm{st}}}$ versus nonclassicality depth *τ*_*M*_ for a bipartition where each part contains three spatial frequency modes for the case of the stimulated noiseless QOFC. The stimulation occurs only in one spatial frequency mode that does not belong to a given bipartition. The amplitude *ξ* of the stimulating field is: *ξ* = 0 (yellow solid curve), *ξ* = 10 (green dashed curve), *ξ* = 50 (blue dotted curve), *ξ* = 100 (violet dash-dotted curve). The total number of spatial frequency modes of the generated QOFC is *N* = 100, and the mean photon number of vacuum fluctuations in each mode is *B*_p,*j*_ ∈ [0, 1].

For a stimulated QOFC, the NI ${E}_{M}^{{\rm{st}}}$ again enhances its sensitivity to detect bipartite entanglement (see Fig. 8). But for larger stimulating fields, the NI ${E}_{M}^{{\rm{st}}}$ becomes less resistant to noise (see Fig. 8). Note that, as in the two-mode case, in order to boost the performance of ${E}_{M}^{{\rm{st}}}$, it is not necessary to stimulate the measured fields. It is already enough to seed only one of all the *N* modes of the QOFC, which does not belong to a given bipartition, in order to make ${E}_{M}^{{\rm{st}}}$ more negative.

## Conclusions

In this study, we have shown the usefulness of the nonclassicality identifier, given in Eq. (14), to detect the bipartite entanglement of the QOFC generated in both spontaneous PDC and stimulated PDC processes. This NI is expressed via integrated second-order intensity moments of the detected optical fields which makes it a convenient and powerful tool for the experimental detection of the entangled modes in QOFCs. We have considered two different cases where a QOFC was comprised either by spatially non-overlapping or completely overlapping frequency modes. We have demonstrated that in both cases the NI displays a good performance in revealing bipartite entanglement for noisy QOFC. Most importantly, with the help of strong stimulating fields, one can sufficiently increase the efficiency of a given NI to reveal the entanglement of QOFCs, but at the expense of a higher sensitivity to thermal noise.
